# A Hybrid Prognostic Method for Proton-Exchange-Membrane Fuel Cell with Decomposition Forecasting Framework Based on AEKF and LSTM

**DOI:** 10.3390/s23010166

**Published:** 2022-12-24

**Authors:** Zetao Xia, Yining Wang, Longhua Ma, Yang Zhu, Yongjie Li, Jili Tao, Guanzhong Tian

**Affiliations:** 1 Ningbo Innovation Center, Zhejiang University, Ningbo 315000, China; 2 School of Information Science and Engineering, NingboTech University, Ningbo 315000, China; 3 College of Control Science and Engineering, Zhejiang University, Hangzhou 310027, China

**Keywords:** prognostics, proton-exchange-membrane fuel cell, hybrid method, degradation prediction, remaining useful life

## Abstract

Durability and reliability are the major bottlenecks of the proton-exchange-membrane fuel cell (PEMFC) for large-scale commercial deployment. With the help of prognostic approaches, we can reduce its maintenance cost and maximize its lifetime. This paper proposes a hybrid prognostic method for PEMFCs based on a decomposition forecasting framework. Firstly, the original voltage data is decomposed into the calendar aging part and the reversible aging part based on locally weighted regression (LOESS). Then, we apply an adaptive extended Kalman filter (AEKF) and long short-term memory (LSTM) neural network to predict those two components, respectively. Three-dimensional aging factors are introduced in the physical aging model to capture the overall aging trend better. We utilize the automatic machine-learning method based on the genetic algorithm to train the LSTM model more efficiently and improve prediction accuracy. The aging voltage is derived from the sum of the two predicted voltage components, and we can further realize the remaining useful life estimation. Experimental results show that the proposed hybrid prognostic method can realize an accurate long-term voltage-degradation prediction and outperform the single model-based method or data-based method.

## 1. Introduction

Owing to the global energy crisis and environmental pollution that humans face, fuel-cell technology has attracted more and more attention from researchers as well as commercial companies. With the advantages of clean, high energy efficiency, and low operating temperature [1,2], the proton-exchange-membrane fuel cell (PEMFC) has been considered as one of the most attractive energy devices for future power applications. However, the durability and the high cost of PEMFC have been the bottlenecks of its large-scale commercial deployment. During operation, the components of the fuel cell, including the proton-exchange-membrane (PEM), the bipolar plate, the gas diffusion layer (GDL), the catalyst layer, and a membrane will degrade due to different working conditions and load cycling [3]. The performance of PEMFC suffers from multiple failure mechanisms, such as conductivity loss, catalyst reaction activity, and mass transfer [4]. The performance of a PEMFC system is characterized by its efficiency and cyclability, which are highly influenced by membrane properties [5]. Shanmugam et al. [6] developed a new block copolymer membrane with a lower self-discharge rate. The cyclability with slight capacity decay showed its chemical stability for long-term operation. Rajput et al. [7] synthesized a graphene oxide composite membrane which has better mechanical and thermal stability. Furthermore, working under highly dynamic conditions, especially in automotive applications, will accelerate the aging process of PEMFC and increase the probability of failure occurrence [8]. The International Energy Agency reported that the cost of commercial fuel cell stack is less than 10,000 USD/kW in 2017 [9]. The maintenance costs drastically decreased from 40 EUR Ct/kWh in 2012 to 20 EUR Ct/kWh in 2017.

The balance of plant (BOP), which mainly consists of an air-supply system, hydrogen-circulation system, water-and-heat-management system and control system, maintains the stable and safe operation of the stack [10]. The degradation mechanism is too complicated to be fully understood with the current technology. To extend the fuel-cell lifetime and reduce its maintenance cost, the management and control strategy of the PEMFC has become a hot research topic. The prognostic method provides a potential solution to extending the PEMFC lifespan [11,12]. As the prerequisite for the maintenance of PEMFC, an effective prognostic method can estimate the state of health (SOH) of the fuel cell and predict the system’s future evolution. By prognostic methods, the degradation process of PEMFC can be investigated and modeled [13] to guide the maintenance services of fuel cells before failures occur. The prognostic methods of PEMFC can generally be divided into three categories [2,14]: the model-based method, the data-based method, and the hybrid method.

The model-based method uses the mechanism degradation model or the empirical degradation model to realize the prognostics of PEMFC. The mechanism model adopts mathematical equations to describe the internal aging process, with the advantages of less training data and strong generality. However, it suffers from a large computational burden and a high complexity of the degradation mechanism [4,14]. Zhang and Pisu [15] built the catalyst degradation model to describe the relationship between operating conditions and the degradation rate of electrochemical surface area (ECSA). Dhanushkodi et al. [16] developed a diagnostic method to characterize the catalyst component durability. Based on the Pt/C catalyst degradation mechanism [17], Polverino and Pianese [18] proposed the dissolution-mechanism model and the Ostwald-ripening-mechanism model to estimate ECSA. However, the validity of the mechanism model needs to be verified by the experimental data, and the adjustment of model parameters depends on expert experience. The empirical degradation model with less computational burden is easier to deploy in online applications. Jouin et al. [19] proposed a PEMFC prognostic method based on logarithmic, polynomial, and exponential empirical equations. Bressel et al. [13] proposed a typical semi-empirical prognostic method that brings polarization curves into consideration. Li et al. [20] proposed an estimation algorithm for lithium-battery SOC in electric vehicles based on an adaptive unscented Kalman filter (AUKF). Zhang et al. [1] realized internal characterization-based prognostics for fuel cells based on a Markov-process algorithm.

Data-based methods can be conducted without considering the complex mechanism of PEMFC and can improve the prediction accuracy as long as sufficient monitoring data are available. Silva et al. [21] developed a long-term prediction model for PEMFC based on the adaptive neuro-fuzzy inference system (ANFIS). The wavelet decomposition is proposed in [22] to improve short-term prediction accuracy. In [23,24], the echo state network (ESN) is adopted for forecasting the degradation process. Ma et al. [25] adopted a long short-term memory network (LSTM) to predict the degradation voltage, which identified the superiority of the LSTM network compared with the relevance vector machine (RVM) and the Elman network. Yang et al. [12] proposed an RUL prediction method for the bearing’s degradation process based on LSTM. However, the data-based method suffers from poor generality in practical deployment and there is a shortage of training data because of the costly and time-consuming PEMFC aging test.

The hybrid method is established by combining the advantages of the model-based method and the data-based method through different strategies [26]. It is usually more accurate and robust than a single method at the cost of a more complex structure and a higher computational burden [2]. Peng et al. [11] realized the RUL estimation for a turbofan engine based on the convolutional neural networks (CNN) and long short-term memory (LSTM) structures. Li et al. [27] used a linear-parameter-varying model to build the virtual stack voltage as the health indicator and the degradation trend was predicted by ensemble ESN. Ma et al. [28] fused the extended Kalman filter (EKF) and LSTM algorithms to realize a more accurate prediction result. EKF is used to estimate the system state and then the prediction of LSTM is regarded as the observation for EKF. Based on ANFIS [21], Liu et al. [26] realized the long-term degradation trend prediction and the remaining useful life (RUL) estimation is achieved by AUKF. The membership function is optimized automatically by a particle swarm optimization (PSO) algorithm. The methods above mainly focus on developing new prediction structures. However, the long-term prediction accuracy of those methods still cannot meet expectations: the prediction effectiveness under automotive load cycling needs to be improved.

The voltage-recovery phenomenon occurs periodically after the characterization test and it significantly influences the prediction accuracy. The investigation into this phenomenon can reveal the aging process of fuel cells and support appropriate maintenance strategies. Jouin et al. [29] combined the global power-aging model and power-recovery model based on the particle filter (PF) algorithm to forecast voltage degradation. Morando et al. [30] used the wavelet filter to decompose the stack voltage into two parts and make predictions based on ESN. With the introduction of the self-healing factor, Kimotho et al. [31] realized the prediction of the voltage-aging process after each characterization. Deng et al. [32] proposd a novel empirical model based on the PF algorithm for the remaining useful-life prediction of a lithium-ion battery. The authors separated the local degradation process from the global degradation process to capture the degradation and regeneration phenomena. Zhou et al. [33] divided the voltage data into stationary and non-stationary sequences. Then, the autoregressive and moving average (ARMA) model and time-delay neural network (TDNN) were utilized to predict the degradation voltage. However, the prediction of those models is not robust or accurate enough, as the voltage-recovery phenomenon possesses strong nonlinearity.

Since the PEMFC degradation-process mechanism has not been fully investigated yet, the model-based method’s prediction accuracy cannot meet expectations. The data-based method cannot give a satisfying prediction with enough long-term forecasting horizon. Moreover, the voltage-recovery phenomenon is still a problem for most of the prognostic methods. Thus, it is of great significance to explore a hybrid method to combine the advantages of those two methods to better predict the PMEFC degradation process. In addition, the parameter-adjustment process requires a lot of manual intervention which is very time-consuming. Therefore, it is meaningful to realize model construction and hyperparameters optimization automatically.

A hybrid prognostic method for PEMFC based on the decomposition forecasting framework is proposed in this paper. Specifically, the original voltage data is decomposed into the calendar aging components and the reversible aging components based on the locally weighted regression method (LOESS). Then, we apply the calendar aging model based on an adaptive extended Kalman filter (AEKF) and the reversible aging model based on LSTM to predict the two voltage components, respectively. In this way, the aging process of the PEMFC, including the voltage-recovery phenomenon, can be better forecasted. The final predicted voltage is derived from the sum of the two predictions, and we can further realize RUL estimation. The main contributions of this paper are summarized as follows:

(1) We establish the decomposition forecasting framework to predict the long-term voltage degradation of PEMFC. After the decomposition by LOESS, we apply the AEKF algorithm and the LSTM neural network to predict those two components, respectively. This framework can combine the AEKF method’s advantage of predicting overall aging trends and the LSTM model’s advantage of strong nonlinear-modeling ability. An iterative structure is adopted to realize the long-term degradation voltage forecasting.
(2) Based on the physical aging model, we develop three-dimensional aging factors to better characterize the fuel cell’s aging state. Considering the voltage-recovery phenomenon, we adopt a sliding-window strategy during the training of the LSTM network to improve the prediction accuracy of the model.
(3) The automatic machine-learning (AutoML) method based on the genetic algorithm is adopted to optimize the hyperparameters of the LSTM network automatically, which can improve the prediction accuracy and training efficiency.

The remaining contents of this paper are organized as follows. In Section 2, the decomposition forecasting framework is introduced, followed by the configurations of the AEKF model and the LSTM network. The prediction results and discussions of our method are presented in Section 3. Finally, the conclusion is summarized in Section 4.

## 2. Methodology

### 2.1. The Decomposition Forecasting Framework

**Figure 1:**
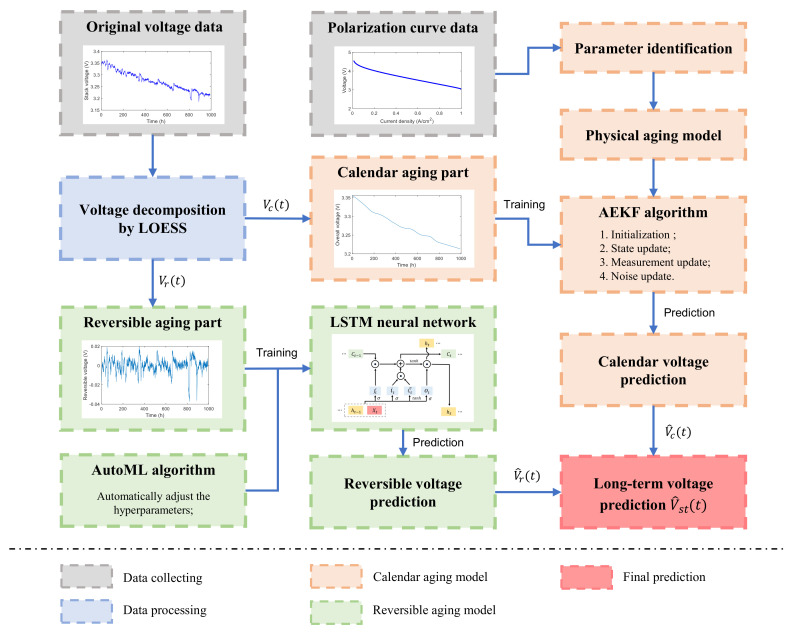
The decomposition forecasting framework of the proposed hybrid method.

The framework of the proposed hybrid prognostic method for PEMFC is shown in Figure 1. First of all, the original voltage data were decomposed into the calendar aging part and the reversible aging part by LOESS. Then, we established the calendar aging model based on the AEKF algorithm to predict the overall aging trend for PEMFC. The genetic algorithm was applied to identify the parameters of physical aging model from the polarization curve. The three-dimensional aging factors were introduced in physical aging model to better depict the degradation trend. Next, based on the LSTM network, we built the reversible aging model to capture the voltage-recovery information. AutoML approach was adopted in the training phase of LSTM for the hyperparameters tuning automatically. In addition, the iterative structure was utilized to realize long-term degradation forecasting [30]. The final prediction of the aging voltage can be obtained by combining the two predicted components and we can further realize RUL estimation.

### 2.2. Dataset Analysis

The dataset we used in this paper comes from IEEE PHM 2014 Data Challenge [34], conducted and collected by FCLAB. The FC1 has a constant current of 70 A while 10% triangular current ripples with the frequency of 5 kHz are added to the 70 A current for FC2. The monitoring data were obtained during the aging test, including voltage, operating parameters, electrochemical impedance spectroscopy (EIS) measurement, and polarization curve. The test bench was adapted for 1 kW fuel cell stack. To master the fuel cells’ running conditions accurately, the experimental operating parameters of the PEMFC can be regulated and measured as shown in Table 1. The gas-humidification subsystem is composed of the two boilers placed upon the stack. Air and hydrogen flow through respective boilers before reaching the stack. Only the air boiler is heated to obtain the required relative humidity. The hydrogen boiler is kept at room temperature due to the need for dry anode gas. The cooling water subsystem dominates the temperature of the stack. The stack voltage is selected as the health indicator of PEMFC degradation since it can be measured easily and it is suitable for online applications [33]. Since the degradation process of fuel cells is slow, the dataset was down-sampled with the interval of one hour to reduce the computational burden. Each considered fuel-cell stack consisted of five cells. The length of FC 1 and FC 2 are 991 h and 1020 h, respectively.

**Figure 2:**
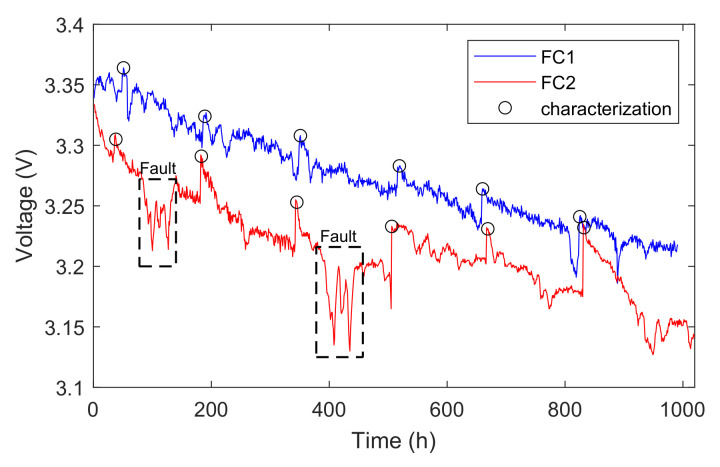
The voltage-degradation curves of FC1 and FC2.

In Figure 2, it is easy to see that the voltage always increases after the characterization test, which is marked by black circles. This is the voltage-recovery phenomenon mainly caused by the interruption of continuous testing during the rest periods [29]. During this time, the water content and distribution of the catalysts return to the previous state, which contributes to ECSA and the proton transfer. The interruption time for characterizations is scheduled weekly, at about 48 h, 185 h, 348 h, 515 h, 658 h, and 823 h for FC1 and 35 h, 182 h, 343 h, 515 h, 666 h, and 830 h for FC2. In addition, it can be noticed in Figure 2 that sudden voltage drops occurred in the dashed boxes, which are regarded as faults during the aging test.

### 2.3. Voltage Decomposition

#### Locally Weighted Regression

Motivated by the idea of decomposition forecasting, the original voltage data is decomposed into the calendar aging part and the reversible aging part by LOESS. LOESS is a nonparametric method for regional regression analysis, which mainly divides the samples into small windows and performs polynomial fitting on them. Repeating this process continuously, we can finally obtain the regression curve. The points near the fitting point have a greater impact on the regression curve and the weight is constructed by the tricube weight function [35]. The weight of fitting points is defined as follows:

(1) $$w_{i}=\left\{\begin{array}{c} {\left(1-{\left|\frac{x-x_{i}}{\Delta (x)}\right|}^{3}\right)}^{3},\left|\frac{x-x_{i}}{\Delta (x)}\right|\leq 1\\ 0,|\frac{x-x_{i}}{\Delta (x)}|\geq 1 \end{array}\right.$$

where Δ(x) is the size of the window, $x_{i}$ is the fitting point, and *x* is the center of the window. The weighted regression can be carried out based on the weighted least-square method.

After the original voltage $V_{st}\left(t\right)$ was filtered by LOESS, we could obtain the calendar aging component $V_{c}\left(t\right)$. Then, the original voltage $V_{st}\left(t\right)$ was subtracted from $V_{c}\left(t\right)$ to obtain the reversible aging component $V_{r}\left(t\right)$. Thus, the fuel-cell stack voltage can be divided into two parts:

(2) $$V_{st}\left(t\right)=V_{c}\left(t\right)+V_{r}\left(t\right)$$

**Figure 3:**
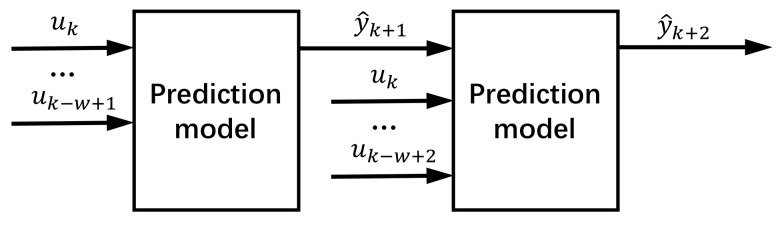
Iterative structure.

As shown in Figure 3, we adopted an iterative structure to realize the long-term time series forecasting [30]. When forecasting *h* steps ahead, we used the value ${\hat{y}}_{k+1}$ just forecasted by a one-step prediction model as part of the input variables for forecasting the next step, where $u_{k}$ represents the input. We continued in this manner until the desired prediction horizon was reached. In particular, prediction errors accumulated through this strategy, which may lead to a divergence in results [36].

### 2.4. Calendar Aging Model Based on AEKF

#### 2.4.1. Physical Aging Model

Previous studies have shown that the polarization curve changes regularly as the operation of PEMFC continues [37], which enables us to build a degradation model based on it. The empirical model of the polarization curve introduced in [13] can be expressed as Equation (3).

(3) $$\begin{array}{cc} V_{c}\left(t\right)= & N\left(E_{ocv}-i\left(t\right)R-aT\ln\left(\frac{i(t)}{i_{0}}\right)\right.\\ & \left.+bT\ln\left(1-\frac{i(t)}{i_{L}}\right)\right) \end{array}$$

where $V_{c}$ is the calendar aging voltage, which represents the approximate part of the stack voltage, *N* is the number of cells, *i* is the stack current, *T* is the operation temperature, *a* is the Tafel constant, *b* is the concentration constant, $E_{ocv}$ is the open-circuit voltage, *R* is the total resistance, $i_{0}$ is the exchange current, and $i_{L}$ is the limiting current.

According to the study in [13], only *R* and $i_{L}$ vary with the operating time obviously during the aging test. Parameters $E_{ocv}$ and $i_{0}$ changed a little, so they can be assumed as constant values. The increase in *R* may result from the polymer membrane’s degradation and the plates’ corrosion [38]. The decrease in $i_{L}$ is related to the ripening of the platinum particles and poor hydrophobicity of GDL, which accounts for the reduction in the mass transfer [39]. Therefore, an aging factor *α* is introduced to describe the change in the aging parameters (*R* and $i_{L}$), since they have similar change speeds [13,26]. The empirical aging parameters can be expressed by Equation (4):

(4) $$\left\{\begin{array}{c} R=R_{0}\left(1+\alpha \left(t\right)\right)\\ i_{L}=i_{L0}\left(1-\alpha \left(t\right)\right)\\ \alpha (t)=\beta t,\beta (t)=\gamma t \end{array}\right.$$

where α(t) represents the degradation state of the fuel cell, β(t) represents the fuel-cell degradation rate, and γ(t) is the derivative of β(t). We notice that a constant β(t) will lead to a linear change in the degradation state α(t), which will reduce the prediction accuracy of the model. Therefore, we introduced another factor, γ(t), so that the degradation rate β(t) can change with time to better forecast the variation in the aging trend. As a result, the three-dimensional aging factors consist of α(t), β(t), and γ(t).

Combining Equations (3) and (4), we can obtain the expression of the physical aging model as follows:

(5) $$\begin{array}{cc} V_{c}\left(t\right)= & N\left(E_{ocv}-R_{0}\left(1+\alpha \left(t\right)\right)i\left(t\right)-aT\ln\left(\frac{i(t)}{i_{0}}\right)\right.\\ & \left.+bT\ln\left(1-\frac{i(t)}{i_{L0}\left(1-\alpha \left(t\right)\right)}\right)\right) \end{array}$$

The degradation process of a fuel cell is nonlinear and can be expressed as Equation (6):

(6) $$\left\{\begin{array}{c} x_{k}=f\left(x_{k-1}\right)+w_{k-1}\\ y_{k}=g\left(x_{k},u_{k}\right)+v_{k} \end{array}\right.$$

where $x_{k}$ is the aging state at *k*th sampling time, $u_{k-1}$ is the input(current), $y_{k}$ is the system output (stack voltage), $w_{k}$ and $v_{k}$ represent the process and measurement noises which are assumed to obey Gaussian distribution with zero mean and variances of Q and R, and f(·) and g(·) are functions used to describe the degradation model.

To better forecast the aging trend of PEMFC, here we introduce three-dimensional aging factors which can be expressed as Equation (7):

(7) $$x_{k}={\left[\alpha_{k},\beta_{k},\gamma_{k}\right]}^{T}$$

where $\alpha_{k}$ is the value of degradation state at *k*th sampling time, $\beta_{k}$ is the degradation rate at *k*th sampling time, and $\gamma_{k}$ is the derivative of *β*. Since $u_{k}=i_{k}$, $y_{k}=V_{c,k}$, the discrete time-state-space equation for PEMFC can be expressed as follows:

(8) $$\left\{\begin{array}{c} \left[\begin{array}{c} \alpha_{k}\\ \beta_{k}\\ \gamma_{k} \end{array}\right]=\left[\begin{array}{ccc} 1 & \Delta t & 0\\ 0 & 1 & \Delta t\\ 0 & 0 & 1 \end{array}\right]\left[\begin{array}{c} \alpha_{k-1}\\ \beta_{k-1}\\ \gamma_{k-1} \end{array}\right]+w_{k-1}\\ y_{k}=N\cdot \left[E_{ocv}-R_{0}\left(1+\alpha_{k}\right)i_{k}-aT\ln\left(\frac{i_{k}}{i_{0}}\right)\right.\\ \left.+bT\ln\left(1-\frac{i_{k}}{i_{L0}\left(1-\alpha_{k}\right)}\right)\right]+v_{k} \end{array}\right.$$

where Δt represents the sample period. Here, the parameters, including $E_{ocv}$, $R_{0}$, *a*, *b*, $i_{0}$, and $i_{L0}$, need to be identified to initialize our calendar aging model.

In order to avoid overfitting, Akaike information criterion (AIC) can be used to measure the fitting results of the proposed model [40]. In general, AIC can be expressed as:

(9) AIC=2k−2ln(L)

where *k* is the number of parameters and *L* is the likelihood function.

Let *n* be the number of observations and *SSR* represent the sum of the squares of the residuals; then, AIC becomes:

(10) AIC=2k+nln(SSR/n)

(11) $$SSR=\sum {\left(y_{i}-{\hat{y}}_{i}\right)}^{2}$$

AIC criterion is used to judge the goodness and the efficiency of the degradation models with two and three parameters (i.e., degradation state, its first derivative, and its second derivative). It can be seen from the Table 2 that AIC of the degradation model with three parameters is less than that of the model with two parameters. The smaller the AIC value, the better the model performance. Therefore, we chose three parameters to build our degradation model.

We regard the mean value of the state estimation as the optimal state estimate, and the point estimation of the trend components can be calculated by the system output matrix. Therefore, we can combine it with the prediction result of LSTM to obtain the final voltage prediction.

#### 2.4.2. Parameter Identification

We identified the parameters of our calendar aging model from the polarization curve data. Considering the multi-parameters and nonlinearity of the physical aging model, we chose the genetic algorithm to realize the parameter identification [39]. The aging factor $\alpha_{k}$ remains at zero since the polarization curve was measured at the beginning of the operation.

The genetic algorithm first initializes the values randomly, and then it performs selection, crossover, and mutation operations on individuals [41] according to the fitness function $f_{fitness}$. The optimal solution can be obtained through the iteration of the algorithm. The fitness function can be expressed as follows:

(12) $$f_{fitness}\left(E_{ocv},R_{0},a,b,i_{0},i_{L0}\right)=\sum_{k}{\left[V_{c,k}-{\hat{V}}_{c,k}\right]}^{2}$$

where $V_{c,k}$ is the observed voltage and ${\hat{V}}_{c,k}$ is the estimated voltage. $E_{ocv}$, $R_{0}$, *a*, *b*, $i_{0}$, and $i_{L0}$ are the parameters that need to be identified.

#### 2.4.3. Extend Kalman Filter

In this paper, we applied the AEKF algorithm to deal with the nonlinearity of the fuel-cell system and to predict the calendar aging voltage. The traditional Kalman-filter algorithm assumes the process noise and the measurement noise as Gaussian white noise with zero means. However, it is difficult to obtain the statistical characteristics of noise in practice. Therefore, the AEKF method is introduced to correct the variance in those noises adaptively, to reduce the impact of unknown noise [42].

For the iterative calculation of our model, the Jacobian matrix can be obtained by linearizing the system with the first-order Taylor formula [39], as follows:

(13) $$\begin{array}{c} \left\{\begin{array}{c} A=\frac{\partial f\left(x_{k-1}\right)}{\partial x}{|}_{x={\hat{x}}_{k-1}^{+}}\\ C_{k}=\frac{\partial g\left(x_{k},u_{k}\right)}{\partial x}{|}_{x={\hat{x}}_{k}^{-}} \end{array}\right. \end{array}$$

The algorithm of the discrete adaptive extended Kalman filter consists of four steps: initialization, state update, measurement update, and noise update, which are shown as follows:

1. Initialization: ${\hat{x}}_{0}^{+}=E\left[x_{0}\right]$, $P_{0}^{+}=E\left[\left(x_{0}-{\hat{x}}_{0}^{+}\right){\left(x_{0}-{\hat{x}}_{0}^{+}\right)}^{T}\right]$, where $E\left[\cdot \right]$ is the mathematic expectation.
2. State update: ${\hat{x}}_{k}^{-}=A{\hat{x}}_{k-1}^{+}+w_{k-1},P_{k}^{-}=AP_{k-1}^{+}A^{T}+Q_{w}$, where ${\hat{x}}_{k}^{-}$ is a-priori state estimate at step *k*, and $P_{k}^{-}$ is a priori estimate error covariance.
3. Measurement update: $L_{k}=P_{k}^{-}C_{k}^{T}{\left(C_{k}P_{k}^{-}C_{k}^{T}+R\right)}^{-1}$, ${\hat{x}}_{k}^{+}={\hat{x}}_{k}^{-}+L_{k}\varepsilon_{k}$, $P_{k}^{+}=\left(I-L_{k}C_{k}\right)P_{k}^{-}$, where $\varepsilon_{k}=y_{k}-g\left({\hat{x}}_{k}^{-},u_{k}\right)$, $L_{k}$ is the Kalman gain at step *k*, ${\hat{x}}_{k}^{+}$ is a posteriori state estimate at step *k*, $P_{k}^{+}$ is a posteriori estimate error covariance at step *k*.
4. Noise update: $\Pi_{k}=\frac{1}{M}\sum_{i=k-M+1}^{k}\varepsilon_{i}\varepsilon_{i}^{T},Q_{w}=L_{k}\Pi_{k}L_{k}^{T},R_{v}=\Pi_{k}-C_{k}P_{k}^{-}C_{k}^{T}$, where $\Pi_{k}$ represents the mapping variance in error, and *M* represents averaging moving window of size.

### 2.5. Reversible Aging Model Based on LSTM

#### Long Short-Term Memory Networks

**Figure 4:**
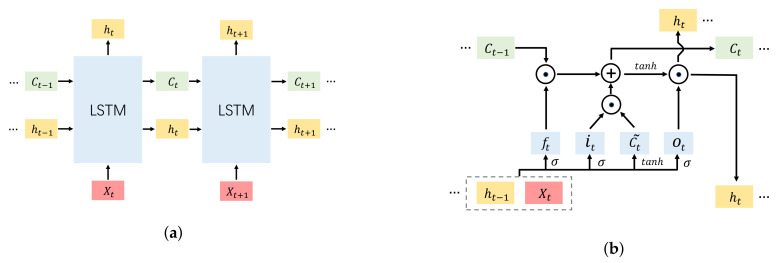
(**a**) LSTM architecture. (**b**) The single cell of LSTM.

Through previous voltage decomposition, we can obtain the reversible aging voltage $V_{r}$ which is the time-series sequence. The recurrent neural network (RNN) has a strong non-linear modeling ability for time-series data, which has achieved great success and wide application in natural language processing (NLP) [43] and time-series problems [44]. With the novel construction of the input gate, the forget gate, and the output gate, the LSTM network can overcome the problem of gradient disappearance or explosion from which traditional RNN suffers [25,35]. The LSTM network is applied to capture the voltage recovery information based on the reversible aging components in this paper. Figure 4a,b illustrates the LSTM architecture and the single cell of LSTM, respectively.

Every time step, the LSTM unit receives the input from the current state $X_{t}$ and the previous hidden state $h_{t-1}$, as Figure 4b shows. The expression of the input gate can be written as:

(14) $$i_{t}=\sigma \left(W_{xi}x_{i}+W_{hi}h_{t-1}+b_{i}\right)$$

The forget gate $f_{t}$ determines which input information should be ignored from the history memory and it is defined as:

(15) $$f_{t}=\sigma \left(W_{xf}x_{t}+W_{hf}h_{t-1}+b_{f}\right)$$

Meanwhile, the candidate value of the memory state ${\tilde{C}}_{t}$ is defined as:

(16) $${\tilde{C}}_{t}=\tanh\left(W_{xc}x_{t}+W_{hc}h_{t-1}+b_{c}\right)$$

Combining Equations (14)–(16), we can obtain the expression to update the cell state:

(17) $$C_{t}=f_{t}⊙C_{t-1}+i_{t}⊙{\tilde{C}}_{t}$$

The output gate $o_{t}$ is responsible for the final output and it is used to update the hidden state $h_{t}$ based on the current cell state $C_{t}$. They can be written as follows:

(18) $$o_{t}=\sigma \left(W_{xo}x_{t}+W_{ho}h_{t-1}+b_{o}\right)$$

(19) $$h_{t}=o_{t}⊙\tanh\left(C_{t}\right)$$

where *σ* is the activation function and we choose the sigmoid function, $W_{xi}$, $W_{hi}$, $W_{xf}$, $W_{hf}$, $W_{xc}$,$W_{hc}$, $W_{xo}$, and $W_{ho}$ are the weight matrices of each gate, $b_{i}$, $b_{f}$, $b_{c}$, and $b_{o}$ are the bias vectors, ⊙ means multiplied by the elements.

The residual components of the voltage data were smoothed by LOESS algorithm again with a window size of 20 to remove random noise or spikes before being sent to LSTM network. After smoothing to remove the noise, the reversible aging voltage data of PEMFCs and time information of characterization tests were input into the LSTM network as features. The network structure consists of four parts: a sequence input layer, an LSTM layer with the maximum number of 300 neurons in the hidden layer, a fully connected layer with one response, and a regression layer. The maximum sliding window size is 300; the loss function is the RMSE; the optimizer is Adam; the epoch size is 200; and the initial learning rate is 0.005.

**Figure 5:**
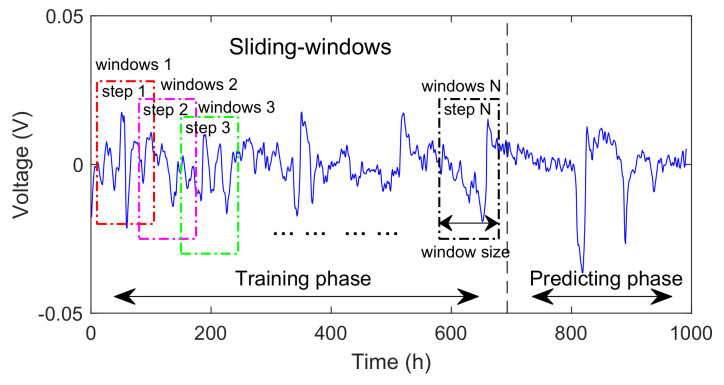
Sliding-window strategy during LSTM training.

We used the reversible voltage data and the time information of characterization from FC1 and FC2 to build samples for our training process of the network. We selected 50%, 70%, and 80% of the sample data as the training set, and the rest was selected as the test data set. The network’s output is the reversible voltage at the next time step. Moreover, as shown in Figure 5, we adopted a sliding-window strategy during the training process of the LSTM. By setting the sliding window size reasonably, we can use the information from multiple times together as the feature input of the LSTM to improve the model’s prediction ability for time-series data.

### 2.6. AutoML Algorithm

The training of neural networks requires a lot of manual intervention which is very time-consuming. Here, with the help of the AutoML algorithm, we can realize model construction and hyperparameters optimization efficiently. Particularly, we apploed the genetic algorithm for finding appropriate hyperparameters of the LSTM network. The genetic algorithm is an optimization method inspired by the evolution process in nature selection [41].

The hyperparameters, including epochs, the number of neurons, and the sliding window size, were initialized arbitrarily ranging from 50 to 400, 50 to 300, and 10 to 300 with different intervals, respectively. Each individual in the population represents a potential solution to the problems to be resolved. The RMSE of LSTM prediction results in the test data set were used as its fitness value. Operations on individuals, including selection, crossover, and mutation, were performed to optimize the population. The parameters of genetic algorithm were set as follows: the population size is 12, the mutation rate is 0.2, the crossover rate is 0.5, and the iteration number is 5. The training process of LSTM was repeated until the genetic algorithm reached its maximum iteration.

## 3. Results and Discussions

The prediction results of our hybrid prognostics method are given in this section. Firstly, the voltage decomposition result is provided and then we will give and discuss our calendar aging model for PEMFC. Combined with the reversible aging model, we will obtain the final prediction.

### 3.1. Voltage Decomposition

**Figure 6:**
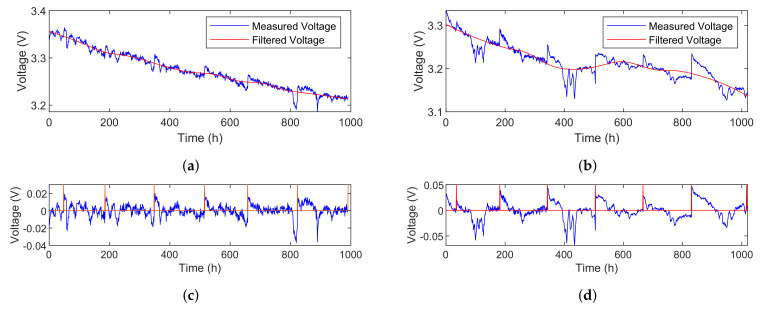
Voltage decomposition result. (**a**) Calendar aging voltage of FC1; (**b**) calendar aging voltage of FC2; (**c**) reversible aging voltage of FC1; (**d**) reversible aging voltage of FC2.

The original voltage data of FC1 and FC2 are decomposed into the calendar aging part and the reversible aging part based on the LOESS, as shown in Figure 6. The smooth window size of FC1 is 300 and since FC2 fluctuates more violently, we set it to 500. The characterization tests (including the polarization curve test and the EIS measurement) are performed once a week, so the voltage recovery phenomenon appears periodically as shown in Figure 6c,d, where the red line indicates whether the characterization test was carried out. We can notice that the degradation of FC2 proves to be faster and more serious than FC1 because of its severe operating conditions.

### 3.2. Calendar Aging Voltage Prediction

Here, we implement the calendar aging model based on the AEKF with the introduction of three-dimensional aging factors (T-AEKF) to better forecast the aging trend for PEMFC. The initial values of the state $x_{0}$, covariance matrix $P_{0}$, process error covariance matrix *Q*, and measurement error covariance matrix *R* were set as follows:

$$\left\{\begin{array}{c} x_{0}={\left[8e-2,2e-4,2e-8\right]}^{T}\\ P_{0}=\left[0.1,0,0;0,0.01,0;0,0,0.0001\right]\\ Q=\left[5e-5,0,0;0,5e-5,0;0,0,5e-5\right]\\ R=100 \end{array}\right.$$

**Figure 7:**
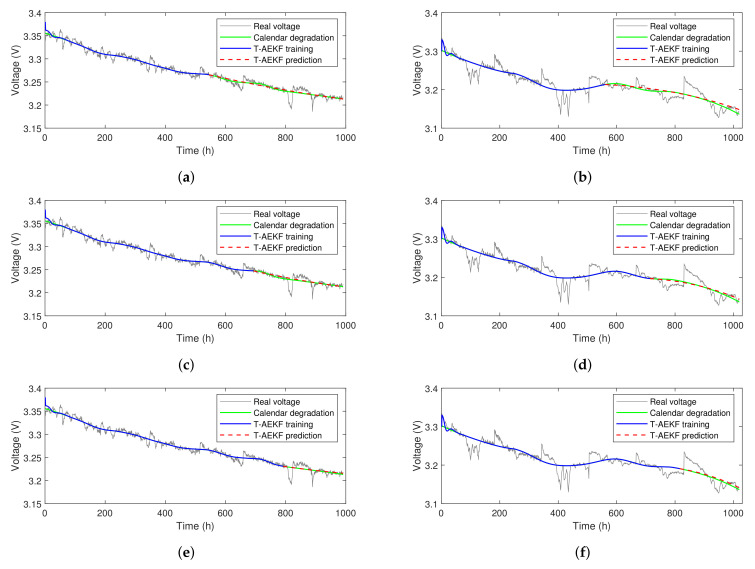
The prediction results based on T-AEKF. (**a**) FC1 with 55% training data; (**b**) FC2 with 55% training data; (**c**) FC1 with 70% training data; (**d**) FC2 with 70% training data; (**e**) FC1 with 80% training data; (**f**) FC2 with 80% training data.

Figure 7a,c,e shows the prediction results based on the T-AEKF for FC1 with 55%, 70%, and 80% training data, respectively. Figure 7b,d,f shows the prediction results based on the T-AEKF for FC2 with 55%, 70%, and 80% training data, respectively. The average values of the aging factors in the training phase were used for the iterative calculation in the predicting phase. The blue lines and the red dotted lines stand for AEKF output values in training and predicting phases, respectively. In Figure 7b,d,f the T-AEKF prediction result of FC2 is slightly higher than the actual value, which can be ascribed to the abnormal voltage drop in the training phase as FC2 worked under more severe operating conditions.

**Figure 8:**
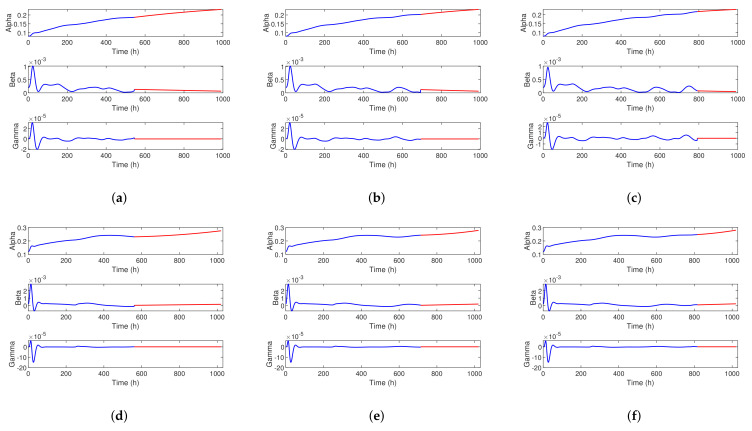
The three-dimensional aging factors of T-AEKF. (**a**) FC1 with 55% training data; (**b**) FC1 with 70% training data; (**c**) FC1 with 80% training data; (**d**) FC2 with 55% training data; (**e**) FC2 with 70% training data; (**f**) FC2 with 80% training data.

Figure 8a–c demonstrates the estimation results of aging factors for FC1 with 55%, 70%, and 80% training data, and Figure 8d–f demonstrates the estimation results of aging factors for FC2 with 55%, 70%, and 80% training data. The blue lines and the red lines represent the aging factors in the training and the predicting phases, respectively. From Figure 8, we can see that the aging factor *α* increases slowly as the *β* decreases with a fluctuation tend. The AEKF algorithm can estimate the aging factor *α* iteratively so as to update the prediction of the voltage. In the predicting phase, factor *γ* remains constant. These results show that with the introduction of three-dimensional aging factors (*α*, *β*, *γ*), the proposed calendar aging model can accurately track the overall aging trend both in training and predicting phases.

Crucially, compared with the real voltage, we note that the spikes and fluctuations exist at the characterization time point for FC1 and FC2 during the aging test, which is regarded as the voltage recovery phenomenon. That is why we built the reversible aging model to capture detailed information for voltage degradation.

### 3.3. Reversible Aging Voltage Prediction

We deployed the reversible aging model to capture detailed information on the voltage recovery phenomenon. After the voltage decomposition, the sequence of reversible aging voltage is fed into the LSTM network, where the output is the reversible voltage at the next time step. Inspired by [25], we implemented the sliding-window strategy to rebuild the data structure and to improve the prediction accuracy. Since the interruption time of characterization tests is known in advance, we input this information into LSTM as one of the features to make a better prediction, as shown in Figure 6c,d. Additionally, for FC2, as the sharp voltage drop in the two blue dashed boxes is not caused by the normal aging process, we smoothed this abnormal data to improve the prediction performance.

A total of 55% and 80% of the data were used for training and the rest of the data was used for testing. The loss function is the RMSE; the optimizer is Adam. The hyperparameters, including epochs, the number of neurons, and the sliding window size, were initialized arbitrarily, ranging from 50 to 400, 50 to 300, and 10 to 300 with different intervals, respectively. Then, hyperparameters were optimized by the AutoML algorithm with the iteration of 5, automatically. The predicted results of the reversible aging voltage superimposed with the calendar aging voltage will be provided in our final prediction, below.

### 3.4. Final Aging Voltage Prediction

We added the calendar aging component and the reversible aging component to obtain our final aging voltage prediction. The iterative structure is adopted to realize long-term degradation prediction [21,30]. The predicted values are used as part of the inputs that are fed into the model for forecasting the next step. To verify the advantages of the proposed T-AEKF-LSTM hybrid method, the traditional AEKF method, the LSTM method, and the improved AEKF method based on three-dimensional aging factors (T-AEKF) were used to make a comparison. For the traditional AEKF method, the initial values were the same as the T-AEKF method introduced in Section 3.2. For the LSTM method, the hidden units, epochs, and the sliding windows size were 50, 200, and 20, respectively, which were obtained by testing the performance of LSTM under different configurations.

**Figure 9:**
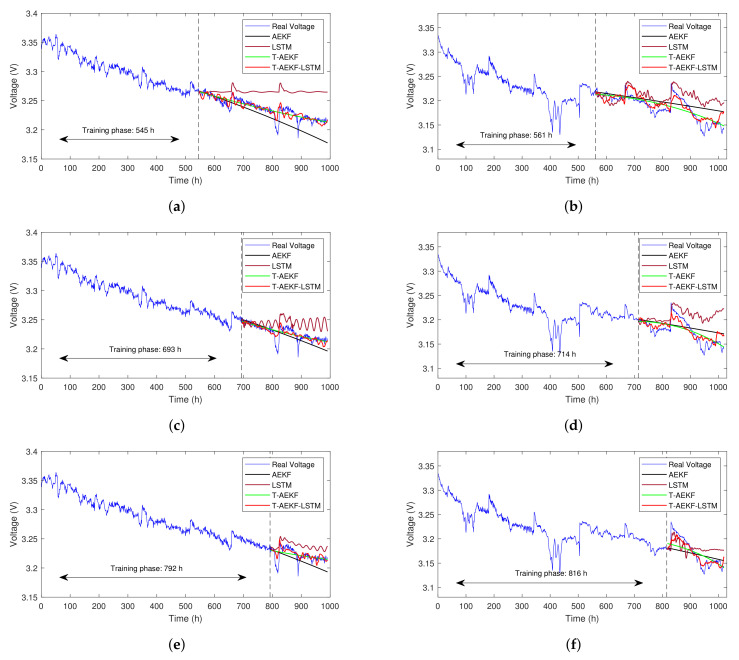
The prediction results of AEKF, LSTM, T-AEKF, and the proposed method (T-AEKF-LSTM). (**a**) FC1 with 55% training data; (**b**) FC2 with 55% training data; (**c**) FC1 with 70% training data; (**d**) FC2 with 70% training data; (**e**) FC1 with 80% training data; (**f**) FC2 with 80% training data.

The predicted results of FC1 under 55%, 70%,and 80% training sets are shown in Figure 9a,c,e, respectively. The traditional AEKF method can only give a linear voltage trend due to its degradation rate remaining constant in the predicting phase. In addition, we find it likely that a bad prediction results when the final point of the training phase is near the abrupt voltage. The LSTM method can predict local nonlinearity which contributes to capturing the voltage recovery phenomenon. However, its output voltage gradually deviates from the measured voltage as time goes on. The T-AEKF method can predict the overall aging trend of PEMFC more accurately than the traditional AEKF method. This decomposition forecasting strategy can prevent the AEKF model from being affected by short-term disturbance and can make the prediction more robust. In addition, three-dimensional aging factors help to model and fit the aging process more accurately, since this scheme can adjust the degradation rate according to the different time. Based on the T-AEKF method and combined with the reversible aging model, the T-AEKF-LSTM method can further capture the voltage recovery information. It can predict the periodic fluctuation in voltage and give a better prediction performance of the aging process for PEMFC compared with other methods.

The prediction results under the dynamic condition for FC2 with 55%, 70%, and 80% training sets are shown in Figure 9b,d,f. It can be found from Figure 9a,d that the AEKF method is not robust enough, as its predicted voltage deviates significantly from the measured voltage. The LSTM method can predict the reversible aging phenomenon after every characterization but fails to trace the aging trend accurately. However, its short-term degradation prediction is more accurate than AEKF and T-AEKF. The T-AEKF can trace the degradation trend better than AEKF but it is not capable of forecasting the reversible aging process. The proposed T-AEKF-LSTM hybrid method can trace the degradation trend and predict reversible voltage components more accurately. It can be noticed that the prediction voltage of the hybrid method will rise slightly at the end of the aging test, which can be ascribed to the memory of the LSTM network, suggesting the occurrence of voltage recovery phenomenon at that time. Thus, the periodic fluctuation in voltage after every characterization test and the nonlinear variation in voltage can be accurately predicted by our hybrid method.

The root mean square error (RMSE) and mean absolute percentage error (MAPE) are used to evaluate the long-term voltage prediction performance [26]. The prediction error is used to evaluate the RUL estimation results. Those criteria are expressed as follows:

(20) $$\mathrm{RMSE}=\sqrt{\frac{1}{N}\sum_{i=1}^{N}{\left(y_{i}-{\hat{y}}_{i}\right)}^{2}}$$

(21) $$\mathrm{MAPE}=\frac{1}{N}\sum_{1}^{N}\frac{\left|y_{i}-{\hat{y}}_{i}\right|}{|y_{i}|}\times 100$$

(22) $$\mathrm{Error}=\mathrm{RUL}-R\hat{U}L$$

where ${\hat{y}}_{i}$ is the predicted voltage, and $y_{i}$ is the measured voltage. RUL represents the actual RUL of the PEMFC, and R$\hat{U}$L represents the estimated RUL.

From the prognostic results for FC1 in Table 3, we can observe that the RMSE and the MAPE of LSTM remain the worst among the four methods. The RMSE and MAPE of T-AEKF are always smaller than AEKF due to the introduction of three-dimensional aging factors as well as the voltage decomposition framework. Since the T-AEKF-LSTM improved the abilities of modeling the reversible aging process based on an LSTM network, it has the lowest prediction error in most cases.

Following the prognostic results for FC2 shown in Table 4, the T-AEKF-LSTM method has the best performance among the three methods according to its lowest prediction error. Particularly, in FC2, the RMSE and MAPE of T-AEKF-LSTM are much lower than that of T-AEKF, while in FC1, the improvements of T-AEKF-LSTM over the T-AEKF method is not very obvious. A possible explanation may be that the voltage recovery phenomenon of FC2 is more severe than FC1 and it greatly reduces the prediction accuracy of EKF-series-based approaches. In contrast, the reversible aging model can capture this detailed information and can significantly improve the prediction performance. The dramatic fluctuation in voltage can also contribute to the training of the LSTM network. The results above show that the proposed hybrid prognostics method can give a more robust and accurate prediction compared with single AEKF or LSTM methods.

### 3.5. RUL Estimation

In this paper, the prediction results of a 55% training set were used to calculate the RUL of PEMFC. The degradation degree 4.0% of the initial voltage was selected as the end of life for fuel cell [33,39]. Since FC2 degrades faster than FC1, 5.0% of the initial voltage was also used to further evaluate the RUL estimation for FC2.

The RUL prediction results based on AEKF, LSTM, T-AEKF, and T-AEKF-LSTM are demonstrated in Table 5. The positive and negative values of the prediction error represent an early prediction or a late prediction, respectively. In order to predict faults in advance, an early prediction is preferred. From Table 5, the RUL estimation error of the proposed T-AEKF-LSTM method is within 30 h and always lower than that of other methods, which indicates that it can give a more accurate RUL estimation among them. The reason for the missing data is that the prediction performances of those methods are too bad to give the prediction errors.

As demonstrated in Table 5, the RUL estimation error of the proposed method is always lower than that of the PAM-ARMA-TDNN method [33] for each degradation degree, which verified the advantages of the proposed method. The results above demonstrate the effectiveness and robustness of the proposed method under static and dynamic operating conditions.

## 4. Conclusions

A robust hybrid prognostic method for PEMFC was proposed in this paper. Considering the voltage recovery phenomenon, a decomposition forecasting framework was established to predict the long-term voltage degradation for PEMFC. Firstly, the original voltage data was decomposed into the calendar aging component and the reversible aging component based on LOESS. Then, we used the AEKF algorithm to predict the overall aging trend of PEMFC based on the calendar aging component. Meanwhile, we introduced three-dimensional aging factors to the physical aging model to better forecast the degradation trend. Next, the LSTM neural network was applied to capture the voltage recovery information through the reversible aging component. Particularly, the AutoML approach based on the genetic algorithm was adopted in the training phase of LSTM for the automatic hyperparameters tuning. The iterative structure was utilized to realize long-term degradation forecasting. The final prediction of the aging voltage can be obtained by combining the two predicted components and we can further realize RUL estimation. We verified the capability of the proposed hybrid prognostic method by two aging datasets under different operating conditions. Experiment results show that the proposed decomposition forecasting framework can combine the advantages of the model-based method for predicting long-term degradation trends and the data-based method for nonlinear modeling ability. In addition, this hybrid method can realize more accurate long-term degradation prediction for PEMFC compared with the single AEKF method or LSTM method. Developing online prognostic methods for PEMFC under high dynamic operating conditions, for example, in automotive applications, is still the major challenge for the prognostic research and it needs further exploration.

## Figures and Tables

**Table 1:** PEMFC stack and experimental operating parameters.

Parameter	Control Range
Number of cells	5
Active area	100 cm^2^
Load current	70 A (FC1)/63–77 A (FC2)
Operating hours	991 h (FC1)/1020 h (FC2)
Air flow rate	23 L/min
Hydrogen flow rate	4.8 L/min
Coolant flow rate	2 L/min
Pressure of anode and cathode	1.3 bar
Stack temperature	55 °C
Relative humidity	50%

**Table 2:** AIC of the degradation models with different parameters for FC1 and FC2.

Stack	Training Data	Numbers of Parameters	
		2	3
FC1	55%	−3677	−4257
	70%	−2687	−2774
	80%	−1792	−1818
FC2	55%	−3564	−3845
	70%	−2347	−2479
	80%	−1545	−1622

**Table 3:** The prognostic results for FC1.

	Data	AEKF	LSTM	T-AEKF	T-AEKF-LSTM
RMSE	55%	0.0181	0.0338	0.0084	**0.0083**
	70%	0.0152	0.0232	**0.0087**	0.0092
	80%	0.0151	0.0188	0.0102	**0.0091**
MAPE	60%	0.4673	0.9101	0.1994	**0.1913**
	70%	0.2792	0.5686	**0.1821**	0.2031
	80%	0.4140	0.5214	0.2162	**0.2039**

**Table 4:** The prognostic results for FC2.

	Data	AEKF	LSTM	T-AEKF	T-AEKF-LSTM
RMSE	55%	0.0201	0.0295	0.0161	**0.0113**
	70%	0.0211	0.0340	0.0169	**0.0126**
	80%	0.0221	0.0314	0.0194	**0.0107**
MAPE	60%	0.5149	0.7690	0.4104	**0.3027**
	70%	0.5716	0.8293	0.4244	**0.3251**
	80%	0.5446	0.8506	0.5226	**0.2640**

**Table 5:** The RUL prediction results for FC1 and FC2 (55% training data).

Stack	Degradation Degrees	Actual RUL	AEKF		LSTM		T-AEKF		PAM-ARMA -TDNN [33]		T-AEKF -LSTM (Ours)	
			RUL	Error	RUL	Error	RUL	Error	RUL	Error	RUL	Error
FC1	4.0%	247 h	216 h	31 h	>446 h	-	283 h	−36 h	252 h	−5 h	**244** h	**3** h
FC2	4.0%	55 h	204 h	−149 h	207 h	−152 h	172 h	−117 h	156 h	−101 h	**29** h	**26** h
	5.0%	359 h	>459 h	-	>459 h	-	386 h	−27 h	381 h	−22 h	**348** h	**11** h

## Data Availability

Not applicable.

## Abbreviations

The following abbreviations are used in this manuscript:

AEKF	Adaptive extended Kalman filter
AIC	Akaike information criterion
AutoML	Automatic machine learning
BOP	Balance of plant
ECSA	Electrochemical surface area
EIS	Electrochemical impedance spectroscopy
FC	Fuel cell
GDL	Gas diffusion layer
LOESS	Locally weighted regression
LSTM	Long short-term memory (neural network)
MAPE	Mean absolute percentage error
PEMFC	Proton-exchange-membrane fuel cell
RMSE	Root mean-square error
RNN	Recurrent neural network
RUL	Remaining useful life
